# The cognitive anchor system: a structural model of self, task selection, and latent intention in human cognition

**DOI:** 10.3389/fpsyg.2026.1763860

**Published:** 2026-07-17

**Authors:** Jiangwei Li

**Affiliations:** Independent Researcher, Guangzhou City, China

**Keywords:** attention control, cognitive architecture, executive function, latent intention, self-representation, structural cognition, task selection, task switching

## Abstract

**Introduction:**

Human cognition requires both stability and flexibility: individuals maintain a continuous sense of self while shifting among tasks, intentions, and contexts. Existing theories explain information processing, prediction, attention, and control, but they leave under-specified the structural layer that determines what cognition is currently organized around.

**Methods:**

This theoretical paper develops the Cognitive Anchor System, a structural framework that distinguishes among the self anchor, the task anchor, and the anchor pool. The model is compared with major theories of attention, intentionality, executive control, predictive processing, working memory, and large-scale neural networks. A minimal boundary function, *y*(β) = (1 − β^2^)^*k*/2^, is introduced to describe how organizational capacity declines as boundary pressure increases.

**Results:**

The framework explains focused attention, task switching, mind wandering, procrastination, decisional conflict, emotional intrusion, creative insight, rumination, and long-term goal maintenance as different configurations of anchor stability, activation, inhibition, and reconfiguration. It generates testable predictions concerning non-linear capacity collapse, collapse-triggered switching, and the continuity of the self anchor under ordinary task competition.

**Discussion:**

The Cognitive Anchor System provides an organizational layer linking self-continuity, task selection, and latent intention. It complements existing cognitive theories and offers empirically tractable directions for behavioral, neurocognitive, and computational research.

## 1 Introduction

Human cognition exhibits a dual nature: it is highly flexible in its moment-to-moment operations, yet remarkably stable in its long-term organization. This flexibility allows individuals to seamlessly shift between tasks—whether solving a logical problem, planning an upcoming commitment, responding to an external cue, or drifting into spontaneous thought—while maintaining a coherent sense of identity and agency. At the same time, the organization of these cognitive shifts is highly stable, governed by an internal structure that guides task selection and prioritization.

While existing theories have significantly advanced our understanding of how cognitive systems organize and shift tasks, they often leave underexplored the structural mechanisms by which cognitive systems determine their focus at a given moment. Traditional theories, such as those relating to attention, executive control, and task switching, explain how information is processed and how tasks are executed, but they typically treat task selection as secondary, presupposing a set of external cues or pre-established routines that guide attention. These models often overlook the critical question: What determines the focus of attention at any given time, and how do latent goals or intentions compete for cognitive priority?

In addressing this gap, the Cognitive Anchor System introduces a structural framework that explains how cognitive systems dynamically shift between tasks and intentions. The system proposes that cognition is organized around three distinct yet interacting types of anchors: the self anchor, the task anchor, and the anchor pool.

1. The self anchor provides the foundational sense of continuity and personal identity across time and task boundaries (Baddeley, 2012).
2. The task anchor defines the current focus of cognitive organization—what the system is engaged with “right now.” This anchor shapes attention, context, and behavioral control (Miller and Cohen, 2001).
3. The anchor pool consists of latent intentions, unfulfilled goals, emotional pressures, and contextual associations, all of which can compete to rise to the level of the task anchor when triggered by specific conditions (Corbetta and Shulman, 2002).

Together, these anchors form the basis for a dynamic, flexible cognitive system that balances the stability of ongoing tasks with the capacity for task switching. Task switching, often perceived as a reactive process, is in fact driven by internal competition between latent goals stored in the anchor pool. When a task switch occurs, it results from a reconfiguration of the anchor hierarchy, shifting focus from one task to another, and this is accompanied by observable neural changes (Dosenbach et al., 2008).

Even during periods of mind wandering, cognition remains anchored to a subjective presence, as the system's underlying anchor configuration continuously influences the direction and content of spontaneous thoughts. This is distinct from externally prompted task switching; voluntary task switching, which occurs when an individual internally decides to change focus, follows systematic patterns driven by the relative activation of different anchors, rather than random fluctuations in attention (Arrington and Logan, 2005). This aspect of endogenous task switching adds a layer of complexity to cognitive flexibility and highlights the role of latent intentions in guiding both thought and behavior.

While existing frameworks describe key cognitive dimensions such as attention and task switching, they do not provide an explicit theory of how tasks are organized at a structural level. The Cognitive Anchor System advances our understanding by framing attention not as a free-floating resource allocation mechanism, but as the stabilization of a task anchor against competition from latent anchors. It also reinterprets mind wandering, procrastination, and intrusive thoughts as natural outcomes of fluctuations in anchor stability, which are influenced by the competition within the anchor pool, rather than simply as failures of executive control.

Furthermore, task switching is framed as a reconfiguration of the anchor structure itself, which reorganizes cognitive priorities, context, and control settings. This structural shift can be seen as the realignment of cognitive resources, providing a more comprehensive understanding of task switching costs and reconfiguration demands. Additionally, the framework explains how long-term goals and emotional states exert influence, even when they are not the current task anchor, by modulating activation levels and thresholds within the anchor pool.

By offering this structural perspective, the Cognitive Anchor System not only integrates existing cognitive theories but also provides a unified vocabulary to reinterpret empirical findings in cognitive neuroscience. Shifts between the default mode network and the multiple demand network can be understood as large-scale manifestations of anchor configuration changes (Corbetta and Shulman, 2002). Prefrontal mechanisms involved in cognitive control can be seen as supporting the stabilization or transition of task anchors. Similarly, emotional intrusions can be viewed as the activation of latent anchors whose thresholds have been lowered by emotional modulation.

Finally, the system makes several testable predictions: cognitive states will differ not only in representational content but also in anchor configuration; task difficulty and emotional arousal should systematically modulate anchor stability; mind wandering frequency should correlate with the activation levels of anchor-pool elements; and neural signatures of task switching should correspond to abrupt reorganizations of anchor hierarchies rather than gradual reallocations of processing capacity (Baddeley, 2012; Dosenbach et al., 2008).

In sum, the Cognitive Anchor System provides a structural foundation for understanding self-continuity, task selection, and the dynamic competition of latent intentions. This framework integrates diverse cognitive phenomena and advances our understanding of cognitive flexibility. The following sections of this paper will further develop the framework, detailing anchor types, mechanisms of anchor transition, and applications to key cognitive behaviors, while also outlining the implications for future research.

## 2 Theoretical background: attention, intentionality, and self in cognitive organization

Human cognition has long been studied through frameworks that emphasize processing mechanisms, representational dynamics, and neural implementation (Anderson, 2013; Miller and Cohen, 2001). However, beneath these explanatory layers lies a crucial structural problem: how is cognitive activity organized at any given moment? Existing theories have provided valuable insights into how cognitive systems organize tasks, but they often leave underexplored the question of how cognition determines its current center of organization, how it decides what is prioritized at a given time, and how it shifts between competing tasks or intentions.

One significant gap in existing theories concerns how cognitive systems shift focus from one task or intention to another. Attention theories have long focused on the mechanisms that allow cognition to prioritize information, arguing that selective processing is necessary because we cannot fully prioritize all competing inputs at once. These theories suggest that attention reflects an interaction between stimulus-driven signals and goal-directed control (Corbetta and Shulman, 2002; Kane and Engle, 2003). Later developments framed attention as a competitive process, shaped by top-down biasing and control mechanisms, often supported by working memory and pre-frontal control processes (Baddeley, 2012; Miller and Cohen, 2001). While these models are successful at explaining how stimuli gain or lose processing priority, they typically assume that the task set or goal state is already established, with “relevance” being pre-defined before attentional selection occurs (Norman and Shallice, 1986). This assumption becomes particularly salient in task switching, where the challenge is not just reallocating attention within a task but also selecting which task set should govern processing in the first place—an issue highlighted by task-switching costs and voluntary task-switching paradigms (Monsell, 2003; Rogers and Monsell, 1995; Arrington and Logan, 2005). In this sense, attention theories provide strong accounts of selection once a task structure is operative, but they do not formalize how competing task-level intentions determine that structure or how latent goals regain influence when they are not actively defining the task.

Intentionality, a concept with deep philosophical roots, refers to the direction of cognition toward objects, goals, or states. In cognitive science, intentionality is often operationalized through constructs such as goal representations, plans, and executive control mechanisms that enable behavior to remain organized around desired outcomes (Miller and Cohen, 2001; Miyake and andand Friedman, 2012). This body of work has generated extensive research into how goals are maintained, monitored, and implemented, including the role of working memory in sustaining task demands (Baddeley, 2012; Kane and Engle, 2003) and the role of planning systems—such as implementation intentions—in translating intention into action (Gollwitzer, 1999). Despite these advances, even within these frameworks, the question of how a specific intention becomes the central organizer of cognition at a given moment—particularly when multiple intentions coexist or when latent intentions intrude—remains implicit rather than explicitly modeled. This problem is also consistent with work on action identification, current concerns, dual-process decision making, and ironic mental control, which shows that action meanings, persistent concerns, automatic evaluation, and suppression processes can reorganize cognitive priority even when they are not the explicit current task (Vallacher and Wegner, 1987; Wegner, 1994; Kahneman, 2011; Klinger, 2013).

The study of self-related processing adds another layer of complexity. Research on self-referential cognition, autobiographical memory, agency, and self-continuity has repeatedly implicated the default mode network in representing aspects of personal identity and internally oriented experience (Buckner et al., 2008; Raichle, 2015). Complementary behavioral and neurocognitive accounts likewise treat the self as a structuring principle that stabilizes experience across time and provides a privileged reference frame for attention and action (Kelley et al., 2002; Sui and Humphreys, 2015). Yet even here, the explanatory emphasis often lies on representational content (e.g., what is encoded about the self) rather than on the organizational structure (i.e., how self-related constraints shape which task becomes dominant and how transitions occur between heterogeneous mental states). As a result, many self-processing theories illuminate the subjective continuity of experience and its neural correlates, but they do not explicitly specify how self-continuity constrains or organizes task-level cognition—precisely the organizational role that the present framework aims to formalize.

These three domains—attention, intentionality, and self—thus converge on a common problem: each presupposes a reference structure that determines which information, goal, or perspective governs cognition at a given moment, but none provides a formal account of that structure. Without such an account, diverse cognitive phenomena risk appearing fragmented rather than unified. For instance, attentional lapses: they are variously attributed to resource depletion, motivational decline, or mind-wandering, yet each interpretation implicitly assumes a shift in underlying task organization and its stabilizing constraints (Baddeley, 2012; Smallwood and Schooler, 2015). Mind-wandering itself is a well-characterized phenomenon that varies systematically with task demands and is linked to measurable costs in performance and wellbeing, suggesting that “lapses” cannot be understood solely at the level of representational content (Killingsworth and Gilbert, 2010; Smallwood and Schooler, 2015). Similar issues arise in procrastination, where a goal of high long-term value is repeatedly displaced by lower-value but more immediately activating alternatives—a pattern widely treated as a robust self-regulatory phenomenon rather than a rare anomaly (Steel, 2007). Indecision can be framed in a related way: competing intentions may hold comparable strength, recruiting executive processes to stabilize one course of action while suppressing others, yet the literature often characterizes the downstream control demands more than the organizational rule that grants dominance to one intention at a time (Miyake and andand Friedman, 2012; Miller and Cohen, 2001). Even sudden insights, often treated as non-linear problem-solving events, can be redescribed as abrupt reorganizations of cognitive priority rather than changes in representational capacity alone.

Taken together, the literature points toward a missing layer of explanation. Attention theories describe how processing unfolds once a task has been selected; intentionality theories describe how goals influence action once they are operative; self theories describe how subjective continuity is preserved across diverse tasks. Yet none explains how cognition selects the organizational center that determines relevance, guides attention, and structures experience at a given moment. This gap becomes especially clear in situations where latent goals suddenly intrude or where emotional states reorganize cognitive priorities without altering environmental demands. These observations suggest that cognitive organization requires a structural model specifying how the system maintains a central anchor, how alternative anchors remain latent, and how transitions among them occur.

The Cognitive Anchor System emerges from the need to articulate such a structural layer. It proposes that cognition is anchored by stable and transient organizing structures that determine the flow of processing, the allocation of attention, and the contextual framing of experience. Rather than treating task selection, intentional direction, and self-continuity as separate problems, the framework integrates them into a unified organizational architecture. Before introducing the full model, it is essential to articulate why these domains require unification and why existing theories lack the structural machinery to achieve it. This section establishes this theoretical motivation: mainstream frameworks effectively describe cognitive operations but do not specify the structural principles that determine what the system is organized around at any given moment. A complete theory of cognition requires such principles, and their absence leaves a conceptual and empirical gap that the present framework seeks to fill.

## 3 The cognitive anchor system: structural definitions

**Figure 1:**
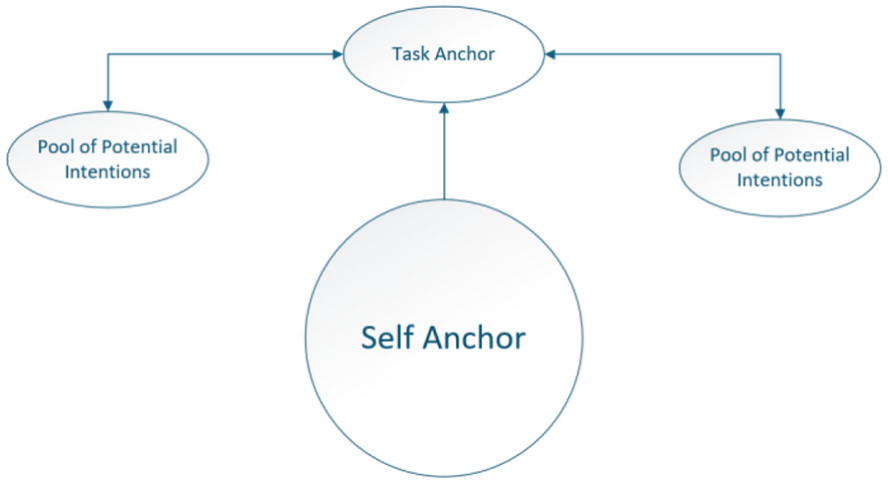
Cognitive anchor system diagram.

To provide a comprehensive explanation of cognitive organization, it is necessary to identify the structural elements that determine what the cognitive system is focused on at any given moment. The Cognitive Anchor System proposes that moment-to-moment cognition is organized around three primary anchors: the self anchor, the task anchor, and the anchor pool. The overall architecture of these three anchors is schematically illustrated in Figure 1. These anchors are not simply representational units or neural entities; they are structural constructs that define the reference points around which cognition is coordinated. By distinguishing and analyzing these anchors, the system provides a foundation for understanding how cognition maintains continuity and flexibility, and how latent intentions influence ongoing activity even when they are not consciously perceived.

It is important to clarify that anchors in the Cognitive Anchor System are not simply goals, knowledge representations, or neural repositories. Rather, they are structural constructs that serve as constraints and provide an organizational framework for cognition. Anchors define the reference points around which cognitive processes are organized and determine how the system prioritizes, selects, and shifts between tasks. For instance, the self anchor is a key organizational structure that centers cognitive activity around a stable, continuous sense of self. It does not refer to a semantic or autobiographical self-concept, but rather represents a structural condition that ensures cognitive activity remains anchored in personal identity across different tasks and time periods. Thus, while goals may change and tasks may shift, the self anchor remains a persistent constraint that governs the organization of thought and behavior, maintaining continuity even during cognitive transitions. This framework of anchors—self, task, and the anchor pool—enables a dynamic, flexible cognitive system that can manage competing priorities and tasks without losing a sense of coherence and direction.

The self anchor is the most stable element in the system, representing the first-person perspective through which cognitive events are experienced as belonging to a single subject. This perspective persists across task switches, emotional fluctuations, and contextual changes, forming a background against which all cognitive activity unfolds. The self anchor is not tied to a semantic self-concept or autobiographical narrative, although these elements contribute to its content. Rather, it serves as the structural foundation that unifies experience and provides continuity across diverse cognitive states.

Above this baseline sits the task anchor, which defines the current focus of cognition. The task anchor specifies the goal, intention, or activity that structures attention, context, and behavior. When active, the task anchor prioritizes cognitive processes that support the current task, while suppressing unrelated processes. The task anchor is responsible for organizing cognitive activity and ensuring its coherence. Though tasks may vary in complexity, duration, and motivational properties, the task anchor functions as a formal structure that organizes processing, regardless of specific task content.

Surrounding the active task anchor is the anchor pool, which consists of latent intentions, unresolved goals, emotional pressures, and contextual associations. These elements do not occupy the center of cognition but retain activation potential and can influence ongoing activity. The anchor pool is dynamic, with elements fluctuating in activation based on context, internal states, and environmental cues. Under certain conditions, elements from the anchor pool can compete with the task anchor, leading to task switching, attentional capture, or intrusive thoughts.

These three anchors—self, task, and anchor pool—interact in a structured hierarchy, with the self anchor providing continuity, the task anchor organizing ongoing activity, and the anchor pool representing a potential for reorganization. The system's organization depends not only on the content of these anchors but also on their activation levels, inhibitory relationships, and competitive interactions. When the task anchor is strongly stabilized, attention remains focused and processing remains coherent. When the task anchor weakens and the activation of latent anchors increases, transitions between tasks become more likely, leading to cognitive shifts such as indecision or mind wandering.

This formal distinction between the anchors helps clarify several important structural principles. For example, the self anchor remains stable during ordinary cognitive transitions, which explains why task switching does not disrupt subjective identity. The task anchor is unique at any given moment; research on multitasking (Arrington et al., 2003) shows that when tasks compete for similar cognitive resources, cognitive performance declines as the system prioritizes one task over another. Finally, the anchor pool contains many potential organizational centers, but only a subset is sufficiently activated to influence ongoing cognition. Activation thresholds determine whether an element of the anchor pool can rise to the level of the task anchor.

A key constraint implied by the anchor view is anchor exclusivity under control-resource overlap: tasks that require overlapping cognitive control settings cannot be concurrently sustained as distinct task anchors without interference. In such cases, the system must serialize execution (rapid alternation) or integrate the demands under a higher-level compound anchor, because attempts at concurrent anchoring force competition for the same control components and degrade performance. Importantly, task similarity can nevertheless facilitate switching: when tasks share more component operations (e.g., attentional control settings or response modality), reconfiguration demands are smaller and switch costs are reduced (Arrington et al., 2003). In the present framework, this corresponds to a shorter “distance” between anchors in a task-control space, lowering the barrier for anchor shifting while still limiting simultaneous anchoring when overlap is high. The framework predicts that high-overlap task pairs should show stronger interference under attempted concurrency, even when switch costs are reduced by similarity.

The interaction of activation and inhibition within the anchor system is critical to understanding cognitive transitions. When the activation level of a latent anchor surpasses the weakening task anchor, task switching occurs. Mind wandering arises when the task anchor's stability decreases, and latent anchors take partial control. Emotional intrusion can be understood as the activation of anchors tied to affectively charged content, while non-emotional cues can influence task priorities by altering activation thresholds within the anchor pool.

The Cognitive Anchor System also explains phenomena like procrastination, where competing anchors influence task priority, and insight, where the system reorganizes cognitive priorities. By framing these phenomena in terms of anchor shifts, the system offers a structural vocabulary that avoids vague constructs like resource depletion or willpower failure, grounding them instead in formal relationships among anchor structures. Furthermore, the system provides a new perspective on focused attention, framing it as the state where the task anchor dominates the cognitive field, suppressing competing anchors.

Finally, the framework clarifies how long-term goals exert influence even when not currently active as a task anchor. These goals remain in the anchor pool, modulating decision-making and task selection without occupying the central position. The system's ability to prioritize and reorganize based on latent intentions provides a structural explanation for commitment, perseverance, and delayed gratification, offering new insights into self-regulation and decision-making processes.

## 4 Mechanisms of anchor switching

The Cognitive Anchor System offers a detailed structural explanation of how cognition transitions from one organizational center to another. This process, known as anchor switching, refers to the shift whereby the dominant task anchor loses its status, and a latent anchor from the anchor pool takes over as the new organizing center of cognitive activity. Unlike representational shifts, which can occur gradually, anchor switching is a structural transition that reorganizes the entire cognitive system, affecting attention, contextual loading, priorities, and behavioral tendencies. This section outlines the mechanisms behind anchor switching, with an emphasis on activation dynamics, inhibitory control, competitive interactions among anchors, and the impact of contextual modulation.

### 4.1 Activation and inhibition mechanisms

Anchor switching is fundamentally driven by the relative activation strength of anchors. Each anchor within the system possesses a specific activation level, which is influenced by both internal and external factors. The active task anchor maintains high activation through goal relevance, motivational support, contextual fit, and executive control. Meanwhile, latent anchors in the anchor pool—such as emotional impulses, unresolved goals, or contextual associations—have varying activation levels depending on factors like their emotional salience, temporal relevance, or long-term significance. A switch occurs when the activation of a latent anchor exceeds that of the task anchor, either due to an increase in the latent anchor's activation, a decrease in the task anchor's activation, or a combination of both. This relational threshold is critical: the cognitive system evaluates anchors not in isolation, but based on their relative activation strength.

However, activation alone does not dictate anchor switching. Inhibition plays a crucial role in maintaining task stability. Inhibitory mechanisms serve to prevent the premature activation of latent anchors that would otherwise challenge the task anchor's dominance. Without sufficient inhibition, minor fluctuations in activation could cause continuous switching, preventing sustained engagement with any task. The strength of inhibition depends on several factors, such as task demands, emotional context, cognitive load, and motivational factors. High-demand tasks tend to recruit stronger inhibition mechanisms, ensuring task stability, while low-demand tasks allow latent intentions to leak into cognitive awareness, increasing the likelihood of switching.

### 4.2 Mathematical model for anchor dynamics

**Figure 2:**
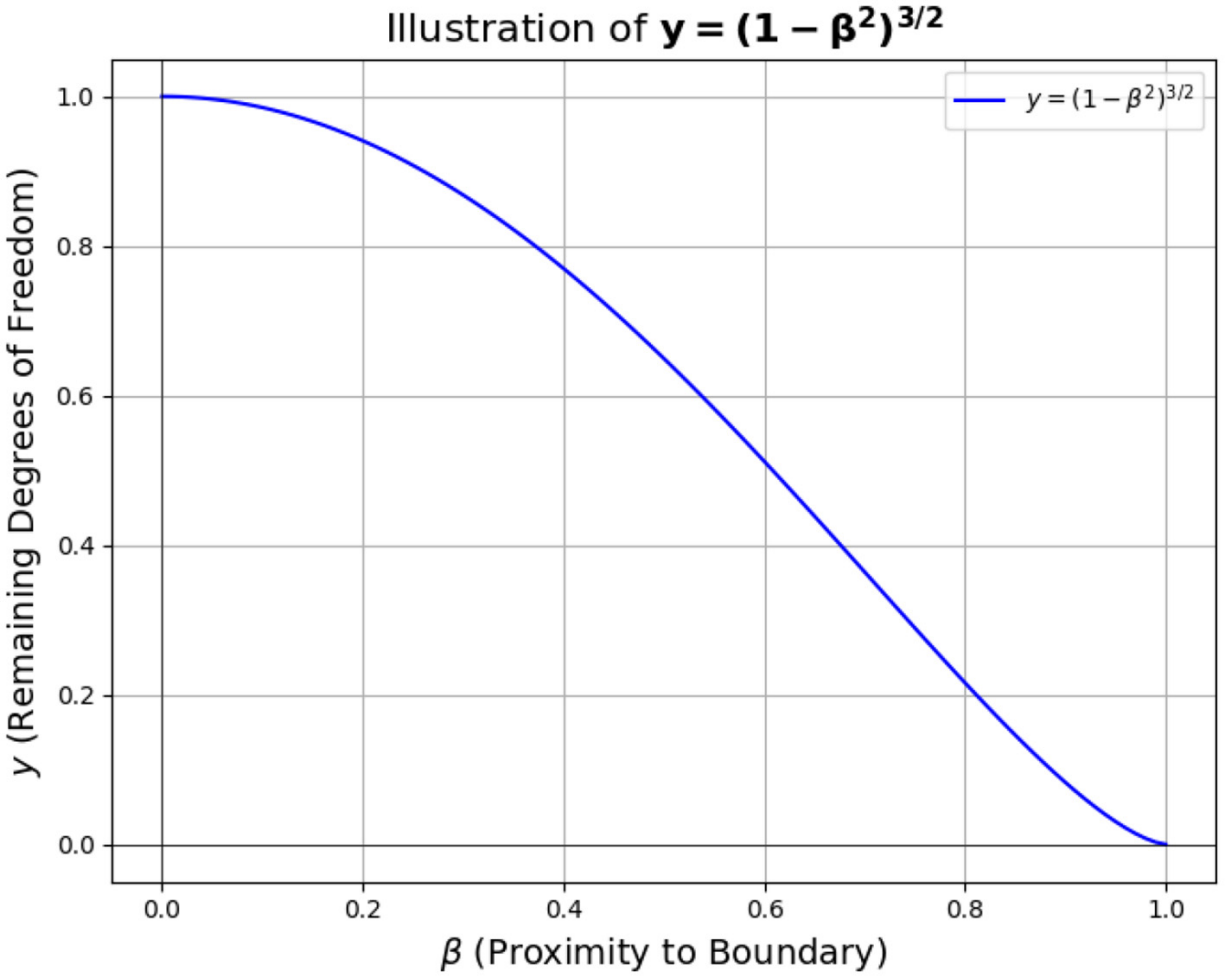
Illustration of the equation *y* = (1 − β^2^)^3/2^ showing remaining degrees of freedom as a function of proximity to boundary (β).

To quantitatively capture these dynamics, we introduce a minimal structural constraint linking the proximity to boundary variable β to the system's organizational capacity y:


$$\begin{array}{l} y\left(\beta \right)={\left(1-\beta^{2}\right)}^{k/2} \end{array}$$

where β ϵ [0, 1], and *k* > 0. In this model, y represents the system's effective capacity to maintain a dominant task anchor—i.e., to preserve task coherence and inhibit competing latent anchors. Figure 2 illustrates the non-linear decay of organizational capacity as β increases toward the boundary. This model serves not as a replacement for mechanistic theories like attention, executive control, or reward processing, but as a concise, falsifiable constraint on how organizational capacity decays as boundary pressure increases toward its limit. The parameter β represents the normalized proximity to the organizational boundary, with the value of *y* decaying as β approaches 1.

For empirical interpretability, β is not a uniquely measurable physical quantity, but rather a normalized proxy capturing how close the system is to its organizational boundary. Depending on the experimental paradigm, β can be operationalized in multiple ways:

- **Demand-to-capacity ratio**: The ratio of current task demand relative to the system's effective capacity to process or integrate information (e.g., working memory load, dual-task interference, rule complexity, or time pressure).
- **Competition-to-stability ratio**: The ratio of competitive pressure from latent anchors relative to the stability of the task anchor. This can be indexed behaviorally through voluntary task switching, intrusion rates, mind-wandering probes, or dwell-time distributions.
- **Control-demand ratio**: The ratio of inhibitory or conflict-control demands relative to available executive resources (e.g., conflict costs or switch costs normalized within participants).

The model predicts that across these different operationalizations, organizational capacity (y) will follow the same non-linear decay function as a function of β, with the rate of decay governed by the exponent *k*. For simplicity, we adopt *k* = 3 as a working default, though further research will be needed to quantitatively estimate *k* across tasks and complexity levels.

### 4.3 Single-anchor collapse

An illustrative example of anchor dynamics can be seen when an individual with basic mathematical training attempts to solve a problem within a fixed task anchor, such as “solve the current math problem.” As the problem's complexity increases—from simple arithmetic to multi-step algebra, and eventually to problems that exceed the individual's cognitive capacity—the system may continue to focus on the same task. However, as boundary pressure increases, the system's organizational capacity begins to deteriorate non-linearly: error clusters increase, volatility rises, and intrusive alternative thoughts (e.g., checking hints, abandoning intermediate steps, or switching to an easier subproblem) become more frequent. This example illustrates a single-anchor regime, where increasing boundary pressure (β) results in an accelerating collapse of organizational capacity (y) without requiring a change in the task anchor itself.

### 4.4 Anchor switching example: job selection decision

Another example of anchor switching can be seen in the decision-making process of choosing between multiple job offers. An individual may face three job options: one with great development potential but low income; another with a good balance of income and stability; and a third with high income but limited career advancement. Here, the individual needs to weigh competing goals—financial stability, career growth, and family needs—each associated with different task anchors.

Throughout the process, the individual remains oriented around the self anchor, which provides a consistent framework for maintaining personal identity and goal alignment. As the individual weighs the pros and cons of each job, they switch between different task anchors: at one moment, they might focus on maximizing income (financial task anchor); at another, they might prioritize life stability or family considerations (stability task anchor); and at other times, they might focus on future career prospects (career task anchor). This example demonstrates anchor switching, where the cognitive system shifts between different organizational centers based on changing goals, contextual influences, and evolving priorities. The switching process is not arbitrary but follows an internal structural mechanism that dynamically adjusts the organizing center.

### 4.5 Conclusion

These mechanisms underscore the structural nature of cognitive transitions. Anchor switching allows cognition to adapt to new demands, maintain continuity across tasks, and integrate internal and external influences. The system's ability to switch between anchors in response to fluctuating goals, emotional salience, and contextual cues provides a more nuanced understanding of cognitive flexibility. By grounding this process in structural relationships among anchors, the Cognitive Anchor System offers a framework for understanding cognitive transitions that complements representational, computational, and neural models. Future research can further validate this framework by testing its predictions in empirical studies of task switching, mind wandering, and goal pursuit.

## 5 Applications to cognitive phenomena

The Cognitive Anchor System provides a structural framework capable of integrating a wide range of cognitive phenomena traditionally explained through disparate theories. By analyzing these phenomena in terms of anchor stability, activation dynamics, inhibition, and switching, the framework unifies behavioral, introspective, and neural observations that otherwise appear fragmented. This section illustrates how the anchor system accounts for key cognitive behaviors, including focused attention, mind wandering, procrastination, conflict, indecision, emotional intrusion, creative insight, and sustained goal pursuit. In each case, the behavior emerges not from isolated processes but from interactions among anchors.

Focused attention is most readily understood through the stabilization of the task anchor. When a task is engaging, meaningful, or sufficiently demanding, strong stabilization occurs through executive mechanisms, contextual coherence, and motivational support. In such cases, latent anchors remain suppressed, and attention remains narrow and directed. The anchor system therefore explains focused attention not merely as efficient resource allocation but as a structural state in which the task anchor maintains dominance over the anchor pool. This perspective clarifies why focused attention is vulnerable to breakdown under conditions of fatigue, emotional disturbance, or prolonged cognitive effort: any reduction in task-anchor stability increases the susceptibility of latent anchors to intrusion. Neural studies showing sustained activation in task-positive networks align with this structural interpretation, reflecting the maintenance of a stable organizing center rather than continuous attentional selection alone.

Mind wandering represents a contrasting cognitive state that emerges when the task anchor weakens and latent anchors gain comparative activation. In this state, elements of the anchor pool exert influence without fully replacing the task anchor, producing fluid transitions among loosely connected thoughts. The structural account reframes mind wandering not as an attentional failure but as a natural consequence of reduced anchor stability. When tasks are routine, low in intrinsic motivation, or internally inconsistent, the task anchor becomes easier to displace. Latent anchors—especially those linked to unresolved goals or emotionally charged content—rise in activation and modulate the stream of thought. This interpretation aligns with empirical findings that mind wandering is more frequent during low-demand tasks, that it correlates with activation in default mode regions, and that it increases with emotional salience of latent intentions. The anchor system therefore provides a principled explanation for why mind wandering varies across contexts and individuals.

Procrastination can be explained as a structural conflict between the long-term value of a task anchor and the immediate activation potential of competing anchors. Tasks associated with long-term benefits often have low immediate activation because they lack strong emotional or contextual triggers. As a result, latent anchors associated with near-term tasks, distractions, or emotionally stimulating activities may dominate the activation landscape despite offering little long-term benefit. Procrastination occurs when these latent anchors repeatedly surpass the activation threshold required to replace the task anchor. The structural account clarifies why motivational interventions sometimes fail: increasing the abstract value of a task does not automatically strengthen its anchoring capacity unless the task also gains activation through emotional, contextual, or short-term relevance enhancements. It also explains why deadlines reduce procrastination by sharply increasing the activation of the pending task anchor and lowering the threshold needed for it to become dominant.

Cognitive conflict arises when two or more anchors possess comparable activation levels without one clearly surpassing the others. This conflict may result from competing goals, conflicting emotional states, or ambiguous task definitions. The structural manifestation is an unstable anchor configuration in which the task anchor lacks sufficient stabilization while multiple latent anchors challenge for dominance. Behavioral indecision, oscillating attention, and subjective tension arise from this competition. Traditional explanations based on limited executive capacity or resource depletion are incomplete because they describe only the consequences of conflict, not its structural origin. The anchor system reframes conflict as a competitive dynamic within the anchor pool driven by insufficient differentiation among potential organizing centers. This interpretation aligns with neural findings that conflict recruits control regions because executive intervention is required to stabilize one anchor or suppress competing anchors.

Emotional intrusion illustrates how affect modulates anchoring directly. Emotional states alter activation thresholds and amplify certain latent anchors by increasing their salience. A distressing memory, perceived threat, or desired outcome may abruptly rise in activation and intrude into ongoing cognition, even when the individual intends to remain focused on the task anchor. Emotional intrusion therefore reflects a structural imbalance in which affectively charged anchors temporarily override inhibitory mechanisms. This explains why emotional content is disproportionately represented in intrusive thoughts and why emotional regulation strategies often work by reinstating inhibitory control or reducing anchor activation. The relationship between emotional states and cognitive organization becomes clearer under this framework: emotions shape the structural landscape of anchors, not merely the content of thought.

Creative insight provides a compelling example of anchor restructuring. During problem solving, repeated application of ineffective strategies stabilizes a rigid task anchor, narrowing the cognitive search space. When this rigidity collapses, suppressed anchors representing alternative perspectives, associations, or solutions may rise abruptly in activation, reorganizing cognitive activity. Insight occurs at the moment when such a previously latent anchor replaces the ineffective task anchor. This structural explanation accounts for the suddenness of insight, the subjective clarity that follows, and the neural signatures showing rapid reconfiguration of activity patterns across task-related networks. Creativity more broadly can be understood as a deliberate weakening of the task anchor to increase the activation potential of latent anchors, enabling the emergence of novel combinations of ideas.

Long-term goal maintenance illustrates how anchors influence cognition outside the immediate focus of attention. Long-term goals rarely serve as task anchors because they are too abstract or temporally remote to structure moment-to-moment cognition. However, they maintain stable but moderate activation within the anchor pool, enabling them to influence decisions and behavior indirectly. When short-term tasks lack compelling activation, long-term anchors may exert greater influence, guiding task selection or shaping deliberation. This structural interpretation explains why individuals often return to meaningful long-term pursuits after periods of distraction and why goal commitment persists despite fluctuations in motivation. Research on goal maintenance and cognitive persistence (e.g., [relevant literature]) demonstrates that even after periods of distraction, individuals can return to their long-term goals, especially when these goals are personally meaningful and align with intrinsic motivation. The activation of long-term anchors, while subtle, plays a critical role in shaping behavior and cognitive direction, even in the presence of competing short-term tasks. It also clarifies how prospective memory tasks depend on the activation of long-term anchors triggered by contextual cues.

The anchor system also illuminates the dynamics of rumination, a phenomenon characterized by recurrent, self-focused, and emotionally charged thought patterns. Rumination arises when a strongly activated emotional anchor repeatedly reclaims the task anchor position despite producing no adaptive benefit. In this configuration, the task anchor becomes cyclically dominated by a particular element of the anchor pool, which remains highly activated due to affective intensity or unresolved conflict. Rumination is therefore a pathological stabilization of an emotionally salient anchor, explaining the difficulty individuals experience in redirecting attention. This model accounts for empirical findings linking rumination to increased activation in self-referential networks and decreased flexibility in control networks.

Decision-making further illustrates anchor interactions. Many decisions require evaluating competing options, each associated with different latent anchors. The difficulty of a decision reflects the relative activation levels of these anchors and the absence of a sufficiently dominant organizing center. Indecision persists when no anchor surpasses the switching threshold. Deliberation itself can be understood as a process of modulating anchor activation through reflection, simulation, and contextual evaluation. When an option's anchor gains sufficient activation, it replaces the competing anchors, yielding a decision. This structural interpretation complements value-based decision theories by explaining the organizational dynamics that precede choice rather than the valuation processes alone.

These applications demonstrate that the anchor system provides a structural vocabulary for cognitive phenomena that are often treated separately. Focused attention, mind wandering, procrastination, conflict, emotional intrusion, insight, long-term goal maintenance, rumination, and decision-making can all be explained as different configurations or transitions among anchors. The framework unifies these behaviors by showing that they arise not from independent mechanisms but from variations in anchor stability, activation profiles, inhibitory processes, and contextual influences. This unification does not replace existing behavioral or neural models but offers a higher-order structure within which such models gain coherence.

Understanding cognition through anchor dynamics also has implications for individual differences. Variability in attention control, emotional regulation, cognitive flexibility, and motivation can be interpreted in terms of differences in anchor stabilization, activation sensitivity, or threshold levels. For example, individuals with highly sensitive emotional anchors may experience frequent emotional intrusion, while those with strong inhibitory control may maintain task stability under distraction. Such structural interpretations could inform clinical research by providing a framework for disorders characterized by impaired anchoring, such as attention-deficit conditions, anxiety disorders, or depression.

By demonstrating explanatory reach across diverse phenomena, the Cognitive Anchor System establishes its utility as a structural theory of cognition. The next section addresses how the framework aligns with and diverges from existing models, including attention theory, predictive processing, control frameworks, and large-scale network accounts.

## 6 Relation to existing models

The Cognitive Anchor System proposes an organizational layer of cognition that complements, but is not reducible to, existing representational, computational, and neural frameworks. In this paper, an *anchor* is not another name for a goal, a task set, or a resource-allocation policy. Instead, it denotes the organizational identity that determines which goal/task-set is currently in force, which alternatives remain latent, and how transitions between them become structurally possible. This distinction matters because many influential theories provide detailed accounts of processing *given* an operative task structure, yet treat the selection and stabilization of that structure as implicit or exogenous. By making this organizational variable explicit, the anchor framework aims to integrate insights across major traditions while sharpening their boundary conditions (Corbetta and Shulman, 2002; Miller and Cohen, 2001; Friston, 2010; Dosenbach et al., 2008; Baddeley, 2012; Smallwood and Schooler, 2015).

Attention and selection. Attention theories explain how processing priority is assigned among competing stimuli and representations via top-down biasing, competition, and control (Corbetta and Shulman, 2002; Kane and Engle, 2003). However, these accounts typically presume that a task set or goal structure already defines what counts as relevant in the first place (Norman and Shallice, 1986). The anchor system formalizes this presupposition by treating relevance as a structural consequence of the task anchor's dominance relative to the latent anchor pool. On this view, changes in motivation, context, or affect alter attention not only by modulating selection signals, but also by shifting the stability of the current anchor and the accessibility of alternatives.

Predictive processing. Predictive processing models provide a unifying account of cognition as hierarchical inference and prediction-error minimization (Friston, 2010; Allen and Friston, 2018). Yet such models often leave implicit which *generative context* is operative when multiple plausible contexts exist. The anchor system specifies how a cognitive system selects and maintains an operative generative context: the task anchor defines the context within which prediction errors are interpreted, while latent anchors correspond to competing contexts that may challenge or replace it. This interpretation reframes contextual reinterpreting, strategy shifts, and switching events as reorganizations of the generative context rather than as changes in inference machinery itself.

Executive control. Executive control frameworks characterize mechanisms supporting goal maintenance, inhibition, conflict resolution, and task switching (Miller and Cohen, 2001; Miyake and andand Friedman, 2012). The anchor system situates these mechanisms within a broader activation landscape: control processes contribute to stabilizing a selected anchor and suppressing competitors, but switching can still occur when the current anchor is structurally weak or when a latent anchor has high contextual or emotional activation. This perspective helps reconcile evidence that switching costs are robust and systematic, while also allowing that some switching events are triggered with limited overt deliberation (Rogers and Monsell, 1995; Monsell, 2003; Arrington and Logan, 2005).

Large-scale networks. Network-level accounts describerecurring configurations among systems such as the multiple-demand network (MDN), default mode network (DMN), and salience network, and emphasize that transitions betweenexternally focused and internally oriented modes are mediatedby switching mechanisms (Dosenbach et al., 2008; Buckner et al., 2008; Raichle, 2015; Sridharan et al., 2008). The anchor frameworkproposes a mapping hypothesis: task-anchor dominance tendsto align with MDN engagement, latent self- and future-oriented anchors with DMN engagement, and anchor transitions with salience-mediated switching. This mapping does not replace network theories; rather, it provides an organizational vocabulary for why particular network configurations recur and why transitions cluster around specific destabilization points (Bressler and Menon, 2010; Spreng et al., 2013).

Working memory. Working-memory models explain how information is actively maintained and manipulated over short timescales and how capacity limits constrain performance (Baddeley, 2012). The anchor framework distinguishes *content maintenance* from *organizational dominance*: working memory may contain representations that are stored without governing cognition, while an anchor may structure cognition even when little content is actively maintained (e.g., intention maintenance across brief interruptions). This distinction helps explain why working-memory load sometimes predicts distraction and switching, but in other cases switching susceptibility is better captured by anchor stability than by representational load alone (Kane and Engle, 2003).

Mind wandering and spontaneous thought. Mind-wandering theories have been framed in terms of executive failure, resource depletion, and spontaneous associative dynamics (Smallwood and Schooler, 2006, 2015). The anchor system reframes mind wandering as structural drift: a weakening task anchor reduces organizational dominance, allowing latent anchors—often autobiographical, affective, or future-oriented—to rise and reorganize cognition. This view is consistent with evidence linking mind wandering to DMN engagement and also explains why spontaneous shifts can sometimes facilitate creativity and problem-solving when latent anchors introduce useful alternative structure.

Summary. Across these domains, the anchor system does not argue against resource limits, inferential dynamics, control mechanisms, or network implementations. Instead, it specifies the structural conditions under which these mechanisms operate: attention theories describe selection once an anchor defines relevance; predictive processing describes inference once a generative context is operative; executive control helps stabilize anchors; network models reflect anchor states and transitions; and working memory supports content maintenance under the currently dominant anchor. By making anchor selection, stability, and switching explicit, the framework aims to unify disparate findings around a single organizational variable and to generate sharper, testable predictions about when and why cognitive organization changes.

## 7 Conclusion and implications

The Cognitive Anchor System offers a comprehensive and structural explanation of how cognition organizes itself, transitions between tasks, and adapts to shifting goals and emotional states. This paper introduces a novel framework that defines cognition not solely as a sequence of representational updates but as an organizational process in which the system dynamically shifts between different task anchors, latent goals, and emotional cues. By formalizing the role of task anchors, self-anchors, and latent anchors, the framework clarifies how cognitive stability is maintained and how flexibility emerges, thereby explaining phenomena like mind wandering, procrastination, creative insight, and emotional intrusion.

The Cognitive Anchor System generates three key testable predictions that can be empirically evaluated without reliance on a specific neural implementation. These predictions present opportunities for theory-testing research and provide a clear path for validating or refuting the framework. By making its assumptions explicit and falsifiable, the present framework also responds to broader calls for stronger formal theory construction in psychology (Oberauer and Lewandowsky, 2019).

The first prediction posits that as boundary pressure (β) increases within a fixed task anchor, there will be a non-linear collapse in organizational capacity (*y*), accompanied by escalating instability. This collapse would manifest through increased intrusion, volatility, or disengagement hazards. A disconfirming pattern would be a linear or shape-invariant relationship between β and stability measures within single-anchor windows, which would suggest that the framework's assumptions about non-linear collapse are flawed. This prediction can be tested using graded difficulty manipulations in fixed-task anchors (e.g., adaptive problem-solving tasks), which should reveal the collapse dynamics of the task anchor under increasing cognitive load.

The second prediction involves the idea that as the system approaches a low-stability zone under the current task anchor, the likelihood of switching to an alternative anchor will rise. This prediction suggests that task switching events should be temporally coupled with collapse signals, where instability markers increase prior to the switch and partially stabilize in the post-switch window under the new task anchor. If switching occurs independently of collapse signals, or without subsequent stabilization, this would disconfirm the hypothesis. To test this prediction, experimental paradigms involving voluntary task switching or multi-option decision-making, combined with intermittent thought probes, can be used to observe how the cognitive system transitions between anchors.

The third prediction posits that self-anchor continuity should remain stable despite increases in task switching and multi-anchor competition under non-pathological conditions. If switching leads to a systematic reduction in self-continuity, this would challenge the framework's assumption about the invariance of the self-anchor. To test this, studies can measure self-continuity in task-switching paradigms, particularly examining how self-anchor continuity varies in response to fluctuating task demands.

These predictions can be tested with well-established paradigms that do not require new experimental machinery. For example, graded difficulty manipulations within a fixed task anchor, such as adaptive problem-solving tasks, can probe single-anchor collapse. Voluntary task switching or multi-option decision paradigms, combined with intermittent thought probes, could test collapse-triggered re-anchoring and the decoupling between task-switching and self-anchor continuity. These experimental approaches offer a straightforward means to validate the theoretical claims made by the anchor framework.

An illustrative example of the cognitive anchor system at work can be seen in the decision-making process involved in choosing between multiple job offers. Imagine an individual faced with three distinct job options: one that offers good career development potential but low income, another that offers stability and proximity to home with a moderate income, and a third that provides high income but lacks future career prospects. In this context, each of these options represents a latent anchor that intermittently dominates the individual's deliberative process, shifting attention and reasoning between one option and another. However, the underlying self-anchor—representing the individual's values, priorities, and long-term goals—remains stable throughout the decision-making process.

The anchor framework predicts that the individual will frequently shift focus between the competing options based on shifting priorities, but the self-anchor remains largely consistent in providing a reference for evaluating each alternative. This highlights the distinction between task-anchor switching and the invariance of the self-anchor, demonstrating that the individual's underlying values and long-term goals can maintain continuity, even as attention shifts between the various job options.

The Cognitive Anchor System offers a structural account of how cognition organizes itself across time, how it transitions between tasks, and how latent intentions influence ongoing thought and behavior. By distinguishing among the self anchor, the task anchor, and the anchor pool, the framework clarifies the conditions under which cognitive stability is maintained and the mechanisms through which flexibility emerges. Throughout this paper, the anchor model has provided organizational principles capable of explaining phenomena ranging from focused attention and mind wandering to emotional intrusion, conflict, procrastination, and insight. It has also shown compatibility with major theoretical traditions, offering a higher-order architecture within which their mechanisms can be integrated.

A central implication of this framework is that cognitive organization is not reducible to representational content, neural activation patterns, or executive demands. These components contribute to cognition, but they do not fully determine the structural center of cognitive activity. The anchor system fills this gap by specifying how anchors are stabilized, how activation fluctuations influence priority, and how transitions occur when latent anchors surpass the task anchor. This provides a more comprehensive understanding of cognitive flexibility and stability, as well as the mechanisms behind task switching and goal pursuit.

One of the primary contributions of the anchor framework is its explanation of cognitive flexibility through anchor switching. Emotional states, for instance, modulate anchor activation thresholds, enabling emotional content to intrude into cognition and shift task priorities. This insight offers a novel perspective on why emotions can disrupt cognitive processes and why emotional regulation is crucial for maintaining stable task performance. The anchor system thus bridges cognitive and emotional theories by highlighting how affective dynamics can structure the entire landscape of cognitive possibilities.

The framework also provides a structural interpretation of context sensitivity. It shows that environmental cues influence cognition not by directly altering representational content, but by modulating activation levels within the anchor pool. This understanding explains why certain cues have different effects depending on context: for instance, a reminder of an obligation may trigger immediate task switching when the task anchor is weak, but may be ignored when the task anchor is strong. Similarly, long-term goals exert influence through latent anchors, guiding decision-making even when these goals do not occupy the central cognitive space.

The framework offers new insights into mental health and pathologies. Disorders characterized by intrusive thoughts, rumination, and compulsive behavior may reflect hyperactivated anchors that dominate the cognitive landscape, preventing the individual from maintaining focus on the task at hand. Anxiety disorders, for instance, may involve reduced inhibitory control combined with heightened activation of threat-related anchors. Depression may manifest as persistently elevated activation of negative self-referential anchors. These insights offer potential pathways for diagnostic conceptualization and the development of targeted interventions.

The Cognitive Anchor System opens up several avenues for future empirical research. Neuroimaging studies could investigate how anchor transitions correspond to changes in brain network activation, particularly within the multiple-demand network (MDN), the default mode network (DMN), and the salience network. Computational modeling can simulate the dynamics of anchor switching, exploring how these transitions lead to stable, unstable, or oscillatory cognitive patterns under varying levels of cognitive load and emotional salience.

Additionally, research could explore how anchor dynamics evolve over the lifespan. For instance, aging could be associated with changes in anchor stabilization, increasing vulnerability to intrusive anchors, or shifts in the balance between long-term and short-term anchors. Longitudinal studies could help us better understand how cognitive control develops and changes with age, and how these changes relate to cognitive decline or adaptations to life transitions.

Finally, integrating the anchor system with computational theories of learning and adaptation, such as reinforcement learning, could further deepen our understanding of how experience shapes cognitive organization across multiple timescales. This integration would help explain how learned policies and reward histories influence anchor activation profiles and guide decision-making processes.

The structural nature of cognition proposed by the Cognitive Anchor System also raises broader philosophical and conceptual questions. If cognition is organized around anchors, then agency, intention, and deliberation can be reinterpreted as operations performed by a system that dynamically selects among competing organizational possibilities. Freedom of action, under this view, is tied to the system's ability to regulate anchor activation, manage conflicts, and sustain or reconfigure its organizational center. This perspective reframes traditional debates about volition and control, suggesting that cognitive autonomy depends not only on representational accuracy or rational evaluation but also on the system's capacity to adaptively organize itself through stable yet flexible anchoring processes.

In conclusion, the Cognitive Anchor System provides a formal and integrative account of how cognition organizes, stabilizes, and transitions between states. It offers a structural explanation for cognitive phenomena such as focused attention, mind wandering, procrastination, emotional intrusion, and insight. The system's emphasis on anchor stability, activation dynamics, and switching mechanisms provides a novel framework for understanding cognitive flexibility and the influence of emotional and contextual factors. By offering a higher-order organizational framework, the Cognitive Anchor System integrates and complements existing theories of cognition and opens new avenues for empirical and computational research, offering a foundation for further investigation into the nature of human thought.
